# Neural network analysis of pharyngeal sounds can detect obstructive upper respiratory disease in brachycephalic dogs

**DOI:** 10.1371/journal.pone.0305633

**Published:** 2024-08-22

**Authors:** Andrew McDonald, Anurag Agarwal, Ben Williams, Nai-Chieh Liu, Jane Ladlow

**Affiliations:** 1 Department of Engineering, University of Cambridge, Cambridge, United Kingdom; 2 Institute of Veterinary Clinical Science, School of Veterinary Medicine, National Taiwan University, Taipei, Taiwan; 3 Queen’s Veterinary School Hospital, Cambridge, United Kingdom; Belgrade University Faculty of Medicine, SERBIA

## Abstract

Brachycephalic obstructive airway syndrome (BOAS) is a highly prevalent respiratory disease affecting popular short-faced dog breeds such as Pugs and French bulldogs. BOAS causes significant morbidity, leading to poor exercise tolerance, sleep disorders and a shortened lifespan. Despite its severity, the disease is commonly missed by owners or disregarded by veterinary practitioners. A key clinical sign of BOAS is stertor, a low-frequency snoring sound. In recent years, a functional grading scheme has been introduced to semi-objectively grade BOAS based on the presence of stertor and other abnormal signs. However, correctly grading stertor requires significant experience and adding an objective component would aid accuracy and repeatability. This study proposes a recurrent neural network model to automatically detect and grade stertor in laryngeal electronic stethoscope recordings. The model is developed using a novel dataset of 665 labelled recordings taken from 341 dogs with diverse BOAS clinical signs. Evaluated via nested cross validation, the neural network predicts the presence of clinically significant BOAS with an area under the receiving operating characteristic of 0.85, an operating sensitivity of 71% and a specificity of 86%. The algorithm could enable widespread screening for BOAS to be conducted by both owners and veterinarians, improving treatment and breeding decisions.

## Introduction

Brachycephalic (short-faced) dog breeds have gained increased popularity due to their anthropomorphic features, perceived good temperament and social media popularity. In the UK in 2021, the French bulldog was the second most popular breed in The Kennel Club with 54,000 registrations [1], and this increased popularity is mirrored worldwide [2, 3]. However, the brachycephalic conformation is associated with a number of diseases, including obstructive breathing, skin fold dermatitis, eye ulceration and prolapse, and spinal disease. Brachycephalic obstructive airway syndrome (BOAS) is common amongst extreme brachycephalic dog breeds, affecting 40% of bulldogs, 50% of French bulldogs and 60% of pugs [4]. BOAS has high morbidity, often presenting at 12–24 months of age and then may progress, resulting in a lifelong impact on quality of life, including exercise intolerance, heat intolerance, gastro-oesophageal reflux, sleep disorders and a decrease in longevity [5, 6].

Clinical signs of BOAS are breed-specific and consist of upper respiratory noises, respiratory effort, reduced nasal airflow, regurgitation, sleep disturbances, cyanosis, overheating and collapse [7]. Unfortunately, BOAS is not easily recognised by owners and can also be accepted as “normal for the breed” by veterinary practitioners. BOAS is a functional disease due to the dynamic components involved in the obstruction or restriction of airflow, and a definitive diagnosis by imaging has not yet been clearly defined [8, 9].

Over the last ten years, a semi-objective clinical respiratory functional grading system (the RFG scheme) for the diagnosis of BOAS has been introduced in the United Kingdom and then internationally, validated using whole-body plethysmography respiratory function assessment in conscious dogs [10, 11]. The RFG scheme is non-invasive, easy to perform, requires no specialist equipment, and is thus useful to screen the dog before breeding in addition to auditing treatment requirements and effectiveness. Dogs assessed to be severely affected (grade 3) require urgent surgical intervention to abate their suffering and prevent suffocation and death, whilst most moderate (grade 2) dogs will improve with either surgical treatment or weight loss [4, 12]. Unaffected or mildly affected dogs (grade 0 or 1, respectively) do not require treatment [4, 12]. The RFG scheme uses criteria based on respiratory noise, effort and cyanosis to classify the overall BOAS grade. Respiratory noise and effort are evaluated before and after an exercise stress test [11]. The respiratory noises are stertor (low-frequency vibratory snoring noise) and stridor (higher-frequency inspiratory noise) and are detected by pharyngolaryngeal auscultation on the side of the neck. A human interpretation is required to classify the noise as mild (only audible with a stethoscope), moderate (constant and audible without a stethoscope) or severe (loud and constant). Stridor has previously been linked to laryngeal disease in humans and dogs [11, 13]. Stertor is similar to the sounds associated with obstructive sleep apnoea in humans [14] and is the predominant clinical sign of BOAS [6, 15]. Its severity is directly linked with the overall grade of BOAS [11].

Human interpretation of respiratory noise requires trained personnel and carries the risk of bias and inaccuracy. An intelligent stethoscope that could automate respiratory noise grading in brachycephalic dogs is desirable to improve the accuracy and accessibility of BOAS diagnosis.

The currently available electronic stethoscopes in the veterinary market are predominately used to cancel noise and record sounds (e.g. eKuore [16]). Whilst some existing devices have machine learning capabilities for human cardiovascular disease [17], they are limited in their classification of respiratory sounds, particularly for veterinary applications. However, ongoing research on applying signal processing and machine learning methods to veterinary sounds is promising. In humans, tracheal and lung sound analysis is repeatable intrasubject [18] and is also useful for identifying the location of disease [14]. The accuracy of respiratory sounds or clinical signs for disease diagnosis in animals is site-dependent, with upper airway sounds being both specific and sensitive for a disease [19]. Neural-network-based sound analysis has been used to distinguish animal call types and (with limited accuracy) classify context for dog barks [20, 21]. Convolutional neural networks have similarly been applied to lung sound classification in human medicine, with promising results reported [22]. The presence of sensor noise, other bodily sounds, and environmental interference can make the analysis of electronic stethoscope recordings challenging, even for a human expert [23]. However, when trained on representative datasets, neural networks can learn to reliably filter out the background noise and recognise complex time-frequency patterns to make accurate and clinically meaningful decisions.

In parallel to this work, Oren et al. [24] design a k-nearest neighbour and decision tree model to detect BOAS using a set of 366 stethoscope recordings from pugs and other brachycephalic breeds. They show promising accuracies at classifying BOAS as either ‘pass’ or ‘fail’ after a fitness test but note that their dataset size is a limiting factor.

This study aims to provide a simple and repeatable method to detect BOAS by developing a machine-learning model to both detect and grade abnormal stertor sounds in brachycephalic dogs. The development and evaluation of a machine learning model relies on a diverse and representative dataset. The key contributions of this work are the creation of a large novel dataset of electronic stethoscope recordings with corresponding respiratory assessment and functional BOAS gradings, and a neural-network-based algorithm that is optimised to detect the time-frequency signature of stertor in the presence of other respiratory sounds and noise.

## Materials and methods

### Dataset

A total of 341 dogs that were presented for airway assessment between September 2015 and September 2021 were included in this study. The most common breed was the French Bulldog (n = 170), followed by Pug (n = 81) and Bulldog (n = 51). The remainder (n = 39) were from other or unspecified breeds. The exclusion criteria was dogs that had lower airway diseases confirmed by images (computed tomography or radiography) or physical examination (thoracic auscultation); dogs that were younger than one year of age (minimal age for RFG scheme); and dogs that had other upper airway diseases (e.g. nasal tumour, trauma) rather than BOAS.

The Department of Veterinary Medicine Ethics and Welfare Committee at the University of Cambridge approved the study and experimental protocol, under ethical review applications CR62, CR63, CR213, and CR215. All dog owners provided informed written consent to include their animals in this study.

The data was gathered at the Queen’s Veterinary School Hospital (Cambridge), Animal Health Trust (Newmarket), breed-specific dog shows, and health clinics. Assessment of each dog was performed by one of five trained veterinarians, following the RFG scheme protocol [10], as part of the dog’s standard clinical assessment. Auscultation with a Littmann 3200 electronic stethoscope (manufactured by 3M Company, Minnesota, USA) was performed over the larynx from the side, with the head in a neutral position. Sound recordings were made for up to 30 seconds with a 4 kHz sampling frequency and 16-bit resolution, using the stethoscopes’ extended frequency range that amplifies sounds from 20 to 2000 Hz. The assessor then graded the severity of stertor and stridor noises as either inaudible, mild, moderate, or severe. The respiratory pattern (regular or irregular) and level of inspiratory effort were also recorded. Regular breathing was defined as breaths that are similar in respiratory rate, tidal volume, and respiratory times. Irregular breathing (seen frequently with upper respiratory breathing obstruction in dogs [4]) was characterized by a mixture of shallow, lower tidal volume breaths with intermittent larger volume breaths.

After the initial recording, each dog underwent an exercise test, which involved 3 minutes of movement at a trotting speed of approximately 4–5 miles per hour at temperatures of 10 to 25 degrees Celsius. No dogs failed the exercise test, which was run by the assessors. Following the test, the respiratory recording and assessment were repeated. The trained veterinarian used the functional grading scheme to assess the overall grading of BOAS from the pre-exercise and post-exercise measurements. Overall, 374 unique encounters were recorded, with a total of 665 individual recordings. A minority of patient encounters (78) occurred after an operation. Study data was collected and managed using REDCap data capture tools hosted at Cambridge University [25, 26].

Table 1 shows the breakdown of the functional grades given to each patient in the dataset. Following the RFG scheme [10], we designate grades 0/1 (none/mild) as “BOAS negative” and grades 2/3 (moderate/severe) as “BOAS positive”. Dogs classified as “BOAS positive” are deemed to be clinically affected by the disease and should be considered for intervention [10].

**Table 1:** Breakdown of study encounters by BOAS functional grade.

		BOAS Functional Grade			
Variable		None	Mild	Moderate	Severe
		(n = 47)	(n = 112)	(n = 157)	(n = 58)
Age		2.3 (1.6–3.6)	2.3 (1.5–3.5)	2.0 (1.3–3.5)	3.1 (1.5–4.1)
Sex	Female intact	9 (19%)	21 (19%)	28 (18%)	9 (16%)
	Female neutered	7 (15%)	23 (21%)	34 (22%)	12 (21%)
	Male intact	10 (21%)	35 (31%)	50 (32%)	23 (40%)
	Male neutered	15 (32%)	25 (22%)	37 (24%)	11 (19%)
Breed	Pug	9 (19%)	31 (28%)	32 (20%)	15 (26%)
	Bulldog	9 (19%)	12 (11%)	26 (17%)	9 (16%)
	French Bulldog	15 (32%)	56 (50%)	88 (56%)	30 (52%)
	Other	14 (30%)	13 (12%)	11 (7%)	4 (7%)
Body condition score	1–3	1 (2%)	3 (3%)	3 (2%)	1 (2%)
	4–6	34 (72%)	80 (71%)	122 (78%)	40 (69%)
	7–9	4 (9%)	20 (18%)	27 (17%)	14 (24%)
Post-op		8 (17%)	34 (30%)	28 (18%)	8 (14%)

Categorical variables are reported as frequency (proportion). Continuous variables are reported as median (interquartile range).

Stertor and stridor are the key audible signatures detected with a stethoscope when assessing the RFG scheme grade. Following the scheme, the presence of moderate or severe stertor or stridor results in a “BOAS positive” grade. However, the prevalence of stertor and stridor in the dataset is not equal. Table 2 shows the number of recordings labelled to contain the different grades of stertor and stridor, demonstrating that the number of moderate and severe stridor recordings is much lower than that of stertor. The table also shows that severe stridor rarely appears without severe stertor. However, the reverse is not true and moderate or severe stertor regularly appears without any stridor. Particularly in the French bulldog and bulldogs, laryngeal collapse, recognisable as stridor, is a consequence of other airway obstruction that produces stertor. Given this clinical reasoning and the make-up of our dataset, neglecting stridor would not have a significant impact on the specificity of our diagnosis as BOAS positive or negative. We, therefore, focus this study on training a machine learning model to detect the presence of stertor sounds.

**Table 2:** Count of recordings with paired stertor and stridor labels.

Stertor	Stridor			
	None	Mild	Moderate	Severe
None	105	7	2	0
Mild	101	35	12	4
Moderate	191	50	8	3
Severe	19	13	5	10

Each table cell shows the number of recordings that have a specific grade of stertor and stridor. For example, 191 recordings were labelled to have moderate stertor and no stridor.

Large machine learning datasets typically hold out around 25 to 30% of the data as an unseen test set to evaluate generalisation and real-world performance. However, the size of the dataset in this study would limit the statistical significance of such a fraction of the data. K-fold cross-validation could be used to estimate the performance of classifiers, but studies have shown that this biases the performance estimate because the model’s hyperparameters are optimised using the test fold [27]. To produce a more accurate estimate of performance, we apply a nested cross-validation strategy, which significantly reduces the bias in the estimation of classifier performance whilst using the entire dataset [27].

**Fig 1:**
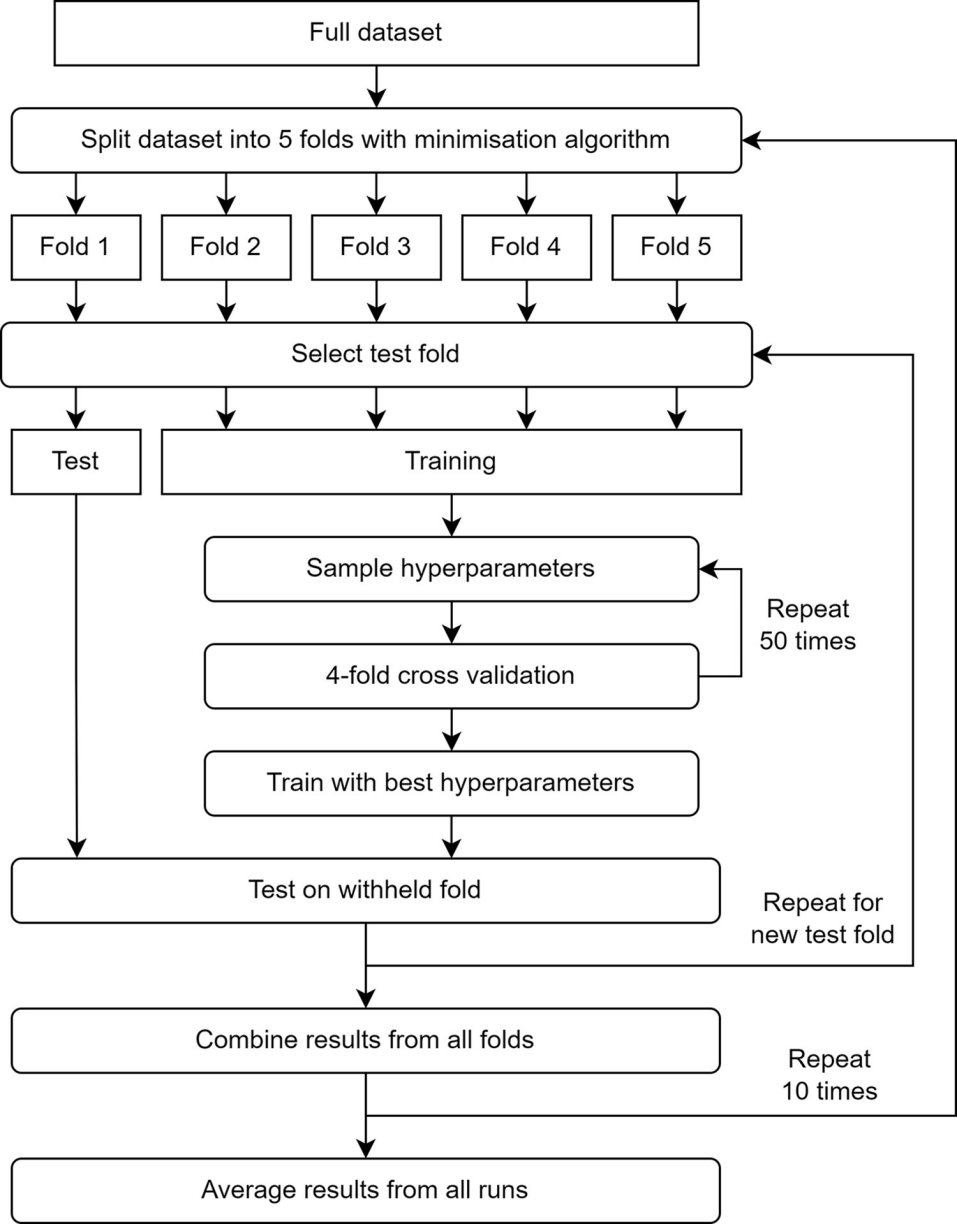
Flow chart showing nested cross validation procedure to train and evaluate models.

Fig 1 details the nested cross-validation procedure, which aims to remove the potential bias introduced by stratifying data into folds, selecting a test portion, and selecting hyperparameters. We first split the data into 5 approximately equally sized folds by applying a stratified minimisation algorithm that balances key prognostic variables (gender, breed, body condition score, BOAS grade) across all the groups. We then pick one fold and remove it as the test fold. We run a four-fold cross-validation on the remaining data, optimising the model hyperparameters using a random search. The best hyperparameters are used to train a single model on the four training folds that then makes a prediction on the unseen test fold. We repeat this four more times, each time with a new test fold. We then repeat the whole process, starting with a new random stratification. This outer loop is repeated 10 times, giving 50 independent runs with their own test predictions and optimised hyperparameters. Finally, we average the performance across all optimised runs when reporting results. This gives an unbiased estimate of how the model would perform on future datasets.

## Methods

To automatically classify the presence of BOAS using electronic stethoscope recordings, we have designed a recurrent neural network (RNN) machine learning algorithm. Audio classification is an active area of machine learning research with several applicable models. Much state-of-the-art research in audio speech recognition uses end-to-end neural network models which directly take a raw audio signal as input and output a prediction. These are often structured as deep convolutional models, such as WaveNet [28], with millions of trainable parameters. Compared to speech recognition, our task is a simpler regression problem but with a much smaller clinical dataset, which limits the applicability of deep models because they would fail to train correctly with few examples. However, we can combine hand-crafted signal processing methods with a smaller-parameter neural network to achieve robust training and accurate predictions.

The key acoustic feature of BOAS evaluated in this work is stertor, an intermittent low-frequency snoring sound due to an elongated and thickened soft palate. Our approach to automatically detect and grade this stertor sound is structured into three main stages. The first is feature extraction, where the audio time series is converted into a reduced complexity representation that a machine learning model can more easily interpret. These features are fed to an RNN trained to predict the grade of stertor (none, mild, moderate, severe) in a single stethoscope recording. Where patients have multiple recordings (e.g. from before and after an exercise test), we then combine predictions to produce an overall BOAS classification (none, mild, moderate, severe) for a patient. All algorithm development is performed in Python, using the SciPy [29] and PyTorch [30] libraries for numerical computing and machine learning models.

### Feature extraction

**Fig 2:**
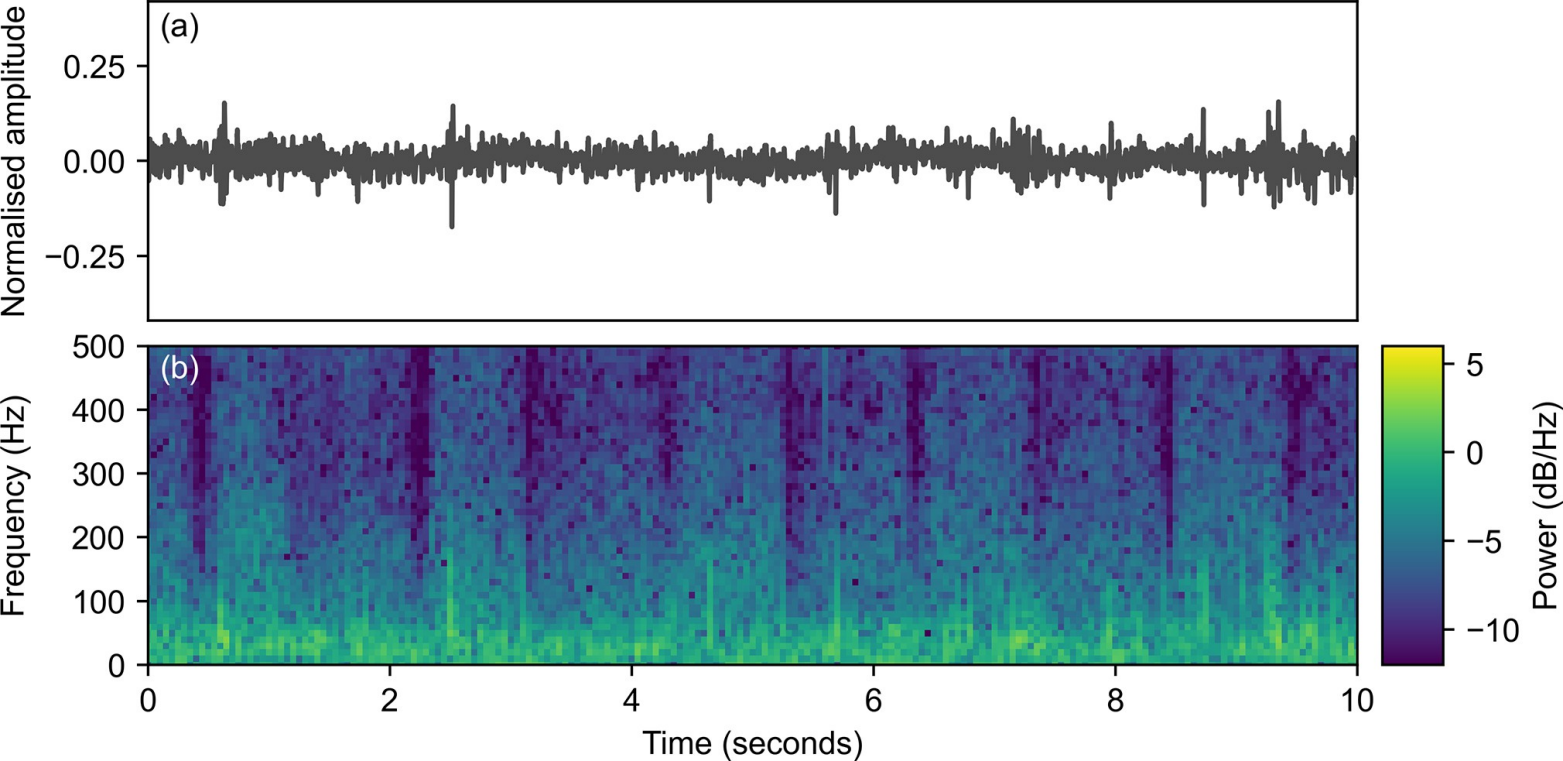
Laryngeal stethoscope recording (a) and corresponding spectrogram (b) for a French bulldog with no audible stertor or stridor. No abnormal sounds are clear in the spectrogram, consistent with the quiet breathing that can be heard in the recording.

**Fig 3:**
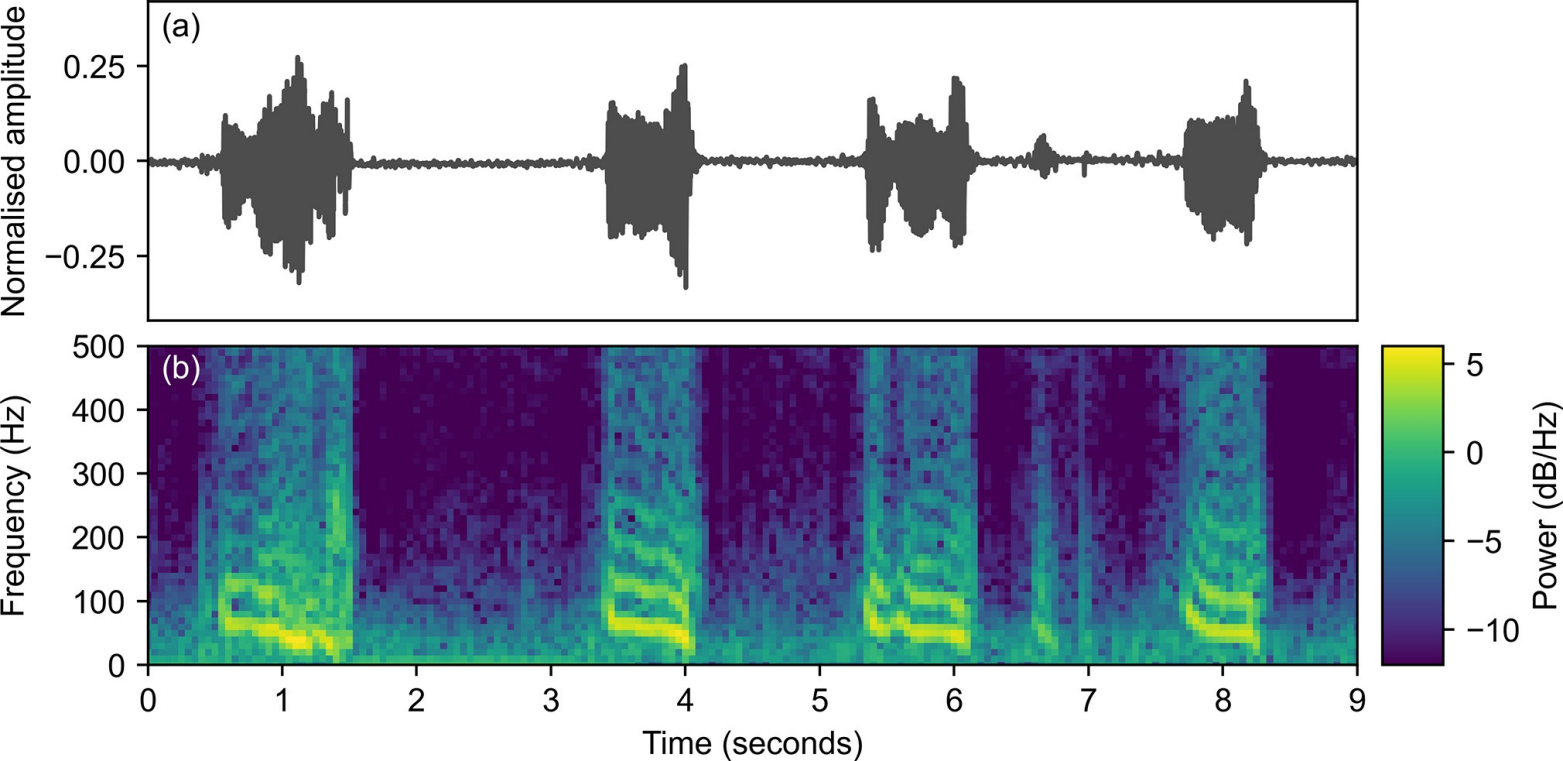
Laryngeal stethoscope recording (a) and corresponding spectrogram (b) for a “BOAS positive” Pug with constant moderate stertor and no stridor. Four distinct stertor sounds are present with fundamental frequencies and harmonics visible in the spectrogram.

We extract spectrograms from the raw audio recordings to create simplified features for the machine learning model. A spectrogram is a two-dimensional representation of a signal, which shows the distribution of the signal’s power in terms of time (x-axis) and frequency (y-axis) [31]. This is useful when analysing how the frequencies in a complex non-stationary recording change over time. Fig 2 shows a stethoscope audio recording and spectrogram from a healthy dog with quiet breathing sounds. In contrast, Fig 3 shows an example recording and spectrogram from a dog with moderate stertor. Here, the stertor can be seen as a strong tonal sound with clear harmonics, with the majority of energy in the signal occurring in low frequencies below 300 Hz.

When extracting our features, the input audio is first normalised to a unit max peak value. The absolute amplitude of the recorded audio is an unreliable feature as it is sensitive to varying application pressures and the location of the recording.

We then extract a spectrogram from the normalised audio time series using a Hann window with a window length of 100 ms and an overlap of 50%. This gives a frequency resolution of 10 Hz, sufficient to localise the position of the stertor in the frequency domain. We then clip the spectrogram to the 20–400 Hz frequency range to limit the vertical size of the features to 39 frequency bins. Whilst the majority of the stertor signal appears to lie below 300 Hz, the expanded frequency range enables the model to use the presence of higher frequency information to distinguish between signal and noise.

### Recurrent neural network regression

The spectrograms in Figs 2B and 3B highlight that the laryngeal recordings are highly non-stationary, with individual stertor sounds in Fig 3 appearing semi-periodically with varying durations. The varying frequency of the stertor sounds makes analysis of fixed frequency tones inappropriate, whilst the level of noise makes it challenging to predict based on analysis of individual time points in the spectrogram. Accurate detection of these sounds, therefore, requires a machine learning model that can learn to model the dependence between individual time points and predict based on sequential analysis of the whole recording. An RNN is a natural model to pick to learn this relationship. Compared to a traditional fully-connected neural network, an RNN includes a hidden state that persists with time so that the predictions for the next timestep are based on both the current and historical features [32].

In our specific application, we use a bidirectional recurrent neural network with Gated Recurrent Unit (GRU) cells [33]. The network is termed bidirectional because it includes separate forward and backwards layers. The forward layer iterates through the recording causally, whilst the backwards layer iterates from the end of the recording to the start. In this way, the neural network can learn to use information from the past and future when making a prediction–in a similar way that a human eye would scan a spectrogram before making a decision. After both layers have iterated through the sequence, the forward and backwards hidden states are concatenated and passed to the next section of the algorithm.

As the RNN produces an output for every timestep in the input features, to attain a single overall classification for a recording, we must combine information from multiple time points. Performing a simple time average of the output of the RNN is ineffective because stertor sounds appear semi-periodically in sections of the audio recording. We, therefore, need the model to learn to pick out key sections of the recording and average them together. To do this, we employ a self-attentive layer [34], which takes the RNN output and uses a small, fully connected neural network (with Tanh activations) to attach an attention weight to each timestep [34]. The attention weights are then used to perform a weighted time average of the RNN output, ensuring that silent or uninformative timesteps are given low weight and do not impact the final prediction. After the attentive layer, the averaged output of the RNN is passed to a fully connected neural network with ReLU activations [35], which transforms the output to a reduced-size vector that matches the number of grades of stertor (4) to predict.

**Fig 4:**
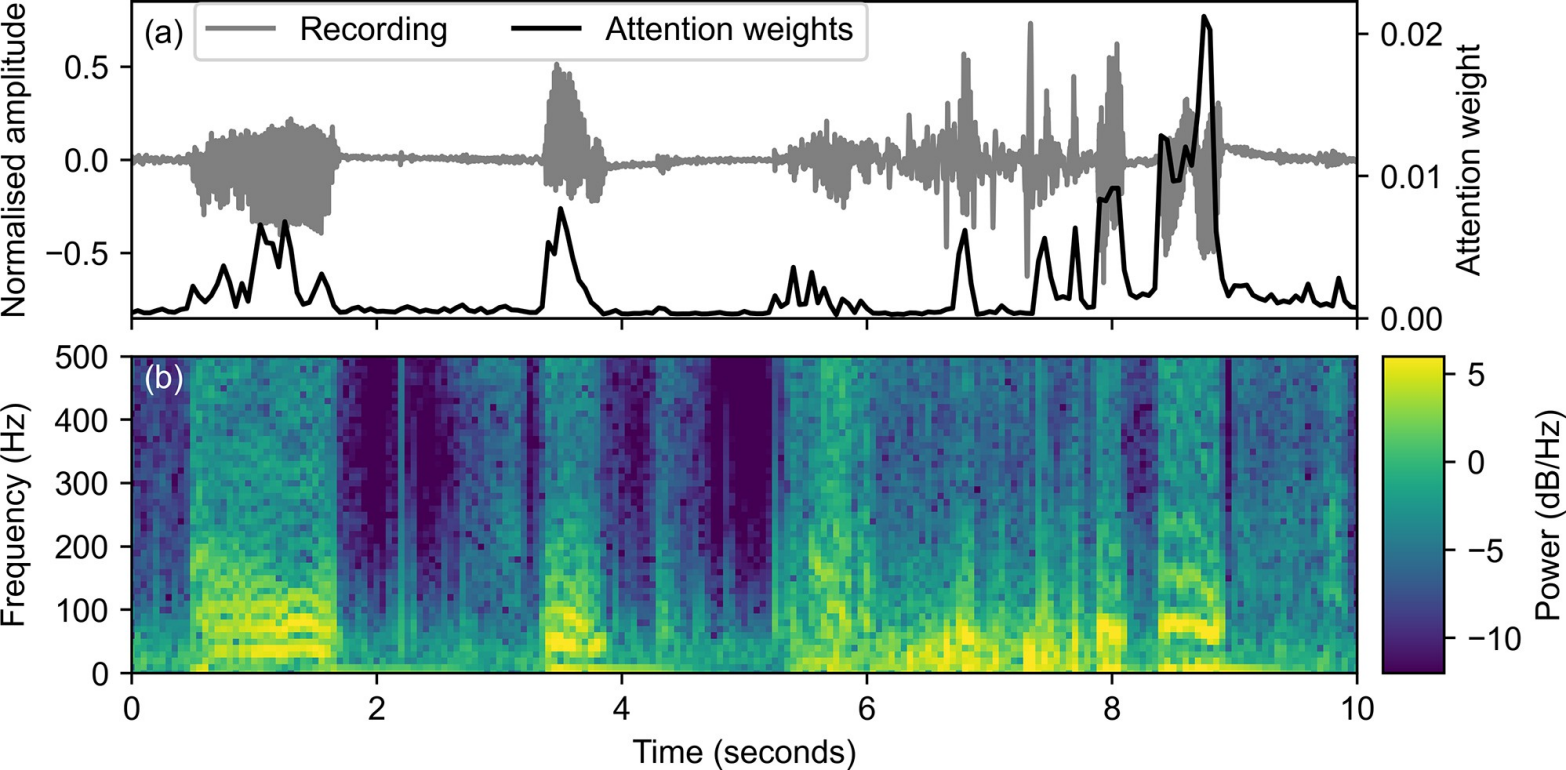
Recurrent neural network attention mechanism weights plotted for a recording (a) with moderate, intermittent nasopharyngeal, stertor. The corresponding spectrogram (b) demonstrates that a combination of time and frequency information is needed to identify the stertor sounds. The RNN in this case is able to correctly use the 2D spectrogram information to address the key stertor sounds and discard silence and noise.

A key advantage of an attention mechanism is that weights predicted by the self-attentive layer provide a visual representation of the timesteps the network is highlighting to make a prediction [34], as shown in Fig 4. The highlighted regions enable easier checking of model errors and provide potentially valuable physical insight for a veterinarian.

The parameters of the RNN and subsequent fully-connected layers (hidden size, number of layers, dropout probability [36]) are chosen as hyperparameters that are optimised during the nested cross-validation scheme. To check if a shorter audio sequence is sufficient to detect stertor, we also test two lengths of input recordings (five or ten seconds) as a hyperparameter.

### Training

The grade of stertor (none, mild, moderate, severe) is a categorical, ordered variable that does not map onto a continuous numerical scale. Automatically predicting the grade is, therefore, an ordinal regression task [37].

A neural network for an N-class classification problem is typically designed to have an output dimension of size N. A softmax function is then applied to convert the output vector of the neural network into a probability mass function. These probabilities are then compared to the one-hot encoded target via a cross-entropy loss function. This method could be applied to an ordinal regression problem, but this would lose the ordering information implicit in the problem (e.g. a mild to severe misclassification is worse than a mild to moderate misclassification). To address this, we follow Cheng’s ‘NNRank’ approach [38]. Instead of a one-hot encoded target vector, the elements for all categories lower than the actual class are also set to 1. We then apply a sigmoid function to each class prediction individually and compare against this target using a binary cross entropy loss. To make a prediction, we set a threshold and choose the lowest category with an output value less than that threshold.

The full model is trained using standard backpropagation techniques and stochastic gradient descent, using the AdamW optimiser [39] with a learning rate of 0.001.

### Combination of predictions

The output of the neural network model for each recording is a single prediction of the stertor grade (none, mild, moderate, severe). We can then directly map the stertor prediction to a BOAS prediction, since in the RFG scheme there is a one-to-one relationship (e.g. moderate stertor results in a moderate BOAS grade). To predict an overall BOAS grade for a patient, information from multiple recordings (generally pre-exercise and post-exercise tests) must finally be combined. We experiment with taking either the maximum or mean of a patient’s predictions and find that the mean result consistently gives marginally better results. This conflicts with the established RFG scheme, where the larger result is used. In this case, taking the mean of results likely acts to regularise the predictions and prevents large spurious estimates from influencing the results.

## Results

**Fig 5:**
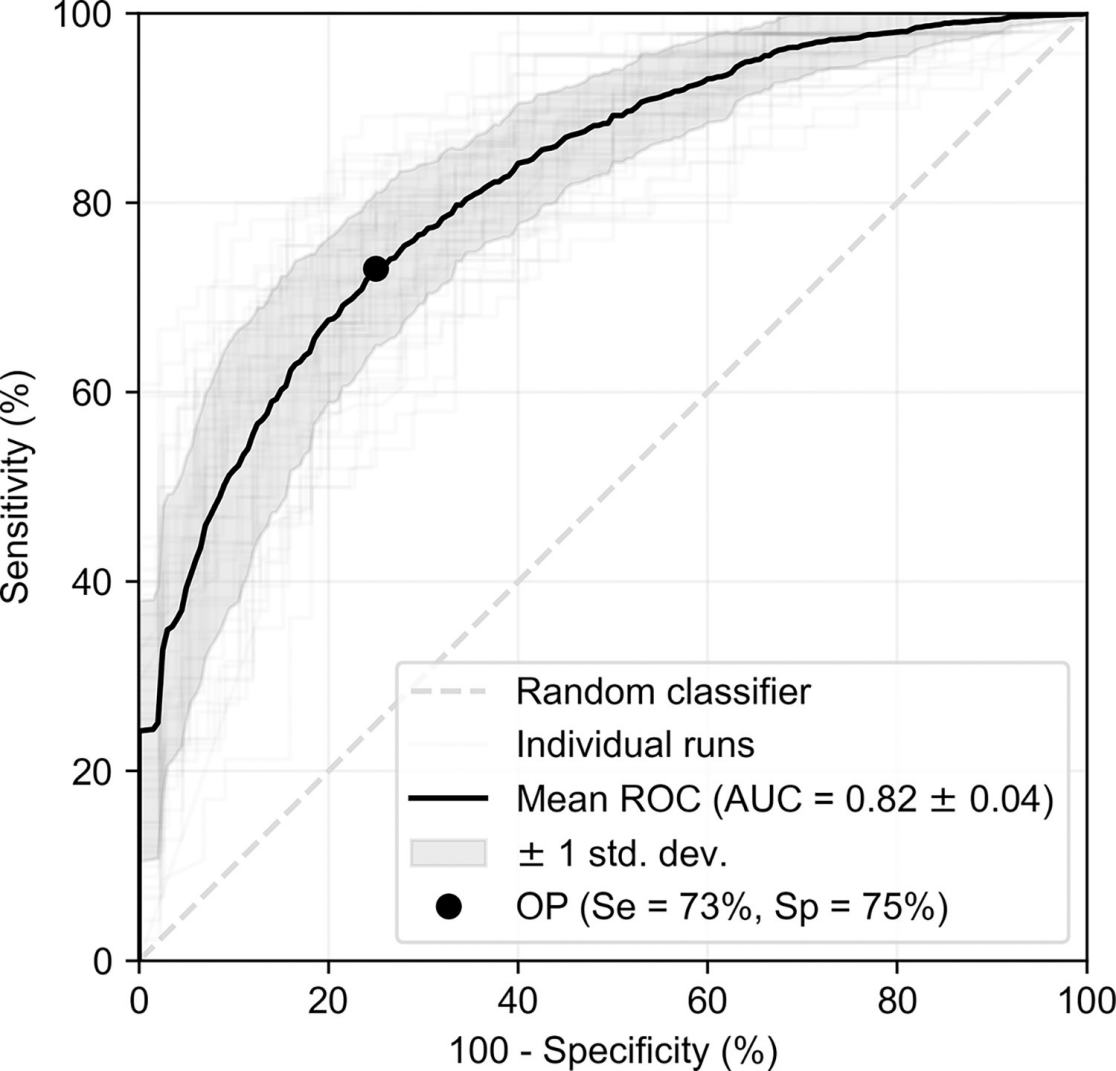
Receiver operating characteristic curves for predicting significant stertor, with the ground truth given by the trained veterinarian’s label. An example operating point (OP) that maximises the sum of sensitivity and specificity is shown.

We first report the performance of the trained models at detecting moderate or severe stertor in each recording. In calculating this result, we group the ordinal regression predictions of the neural network into two classes—none/mild stertor or moderate/severe. We calculate a receiver operating characteristic (ROC) curve for each model produced by the nested cross-validation procedure described in Fig 1, and then calculate a mean ROC plot by applying vertical averaging of individual curves [40]. In this case, we use vertical rather than threshold averaging because the latter assumes predictions of individual classifiers are calibrated [40]. Whilst reporting test results, we excluded patients who had already received surgery to correct their BOAS. This is because the focus of this potential solution is as a screening tool to pick up new cases. The resulting ROC plots are shown in Fig 5. Averaging across all runs, we obtain an area under the curve (AUC) of 0.82 with a standard deviation of 0.04.

**Fig 6:**
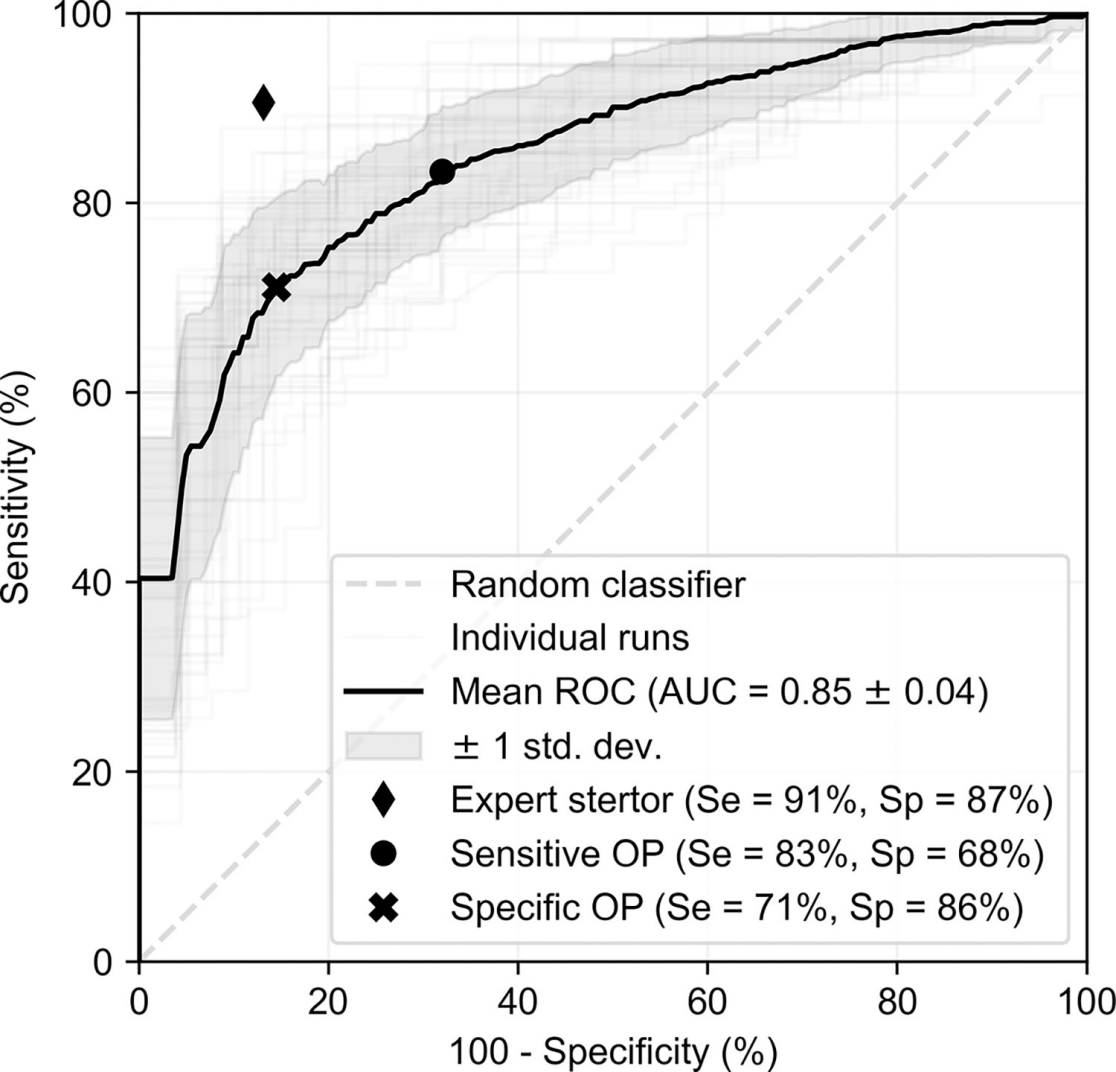
Receiver operating characteristic curves for prediction of ‘BOAS positive’ cases by averaging stertor predictions for a single patient encounter. Two operating points (OPs) for the algorithm are shown which prioritise either sensitivity or specificity. Also shown is the performance of the expert stertor annotation at predicting BOAS, which provides an upper-bound for the algorithm accuracy.

The primary metric we report is the binary prediction of BOAS negative (grade 0 or 1) vs BOAS positive (grade 2 or 3), compared against the ground-truth labels of the expert veterinarian, as shown in Fig 6. Averaging across all runs, we obtain an AUC of 0.85, with a standard deviation of 0.04. If an operating point is chosen to maximise the sum of sensitivity and specificity, the resulting sensitivity is 71%, and specificity is 86%. These are well within the range of accuracy numbers reported in existing screening tests in humans [41]. Also shown in Fig 6 is a sample high-sensitivity operating point, with a sensitivity of 83% and a specificity of 68%. If the ground-truth stertor label (provided by the trained veterinarian) was solely used to predict the final BOAS grade, the result is a sensitivity of 91% and a specificity of 87%. This provides an upper limit on the performance of our neural network approach because the model is trained to replicate the stertor predictions.

Given that the neural network model produces ordinal regression predictions, we can also provide per-class prediction results. Table 3 shows the precision, recall and F1 score for the models, evaluated by averaging individual runs (as was done for the ROC curve in Figs 3 and 4).

**Table 3:** Per-class precision and recall results.

Class	Precision (%)	Recall (%)	F1-score (%)	Support
0 (None)	26.9 ± 36.0	14.5 ± 20.2	16.8 ± 21.5	7.8 ± 1.3
1 (Mild BOAS)	43.9 ± 6.70	78.3 ± 11.2	55.9 ± 7.00	15.6 ± 2.0
2 (Moderate BOAS)	63.5 ± 6.40	55.3 ± 8.10	58.7 ± 5.80	25.8 ± 2.0
3 (Severe BOAS)	52.6 ± 23.9	31.7 ± 16.6	36.6 ± 16.0	10.0 ± 1.3

Each element in the table shows the mean and standard deviation computed by averaging all individual model results.

## Discussion

This work focuses on identifying clinically significant BOAS using the audible markers of stertor in stethoscope recordings. Fig 5 demonstrates reasonable performance at distinguishing significant stertor in a single audio recording (AUC 0.82), but discrepancies with the expert labeller are still common. As described in the RFG scheme, one distinguishing feature of moderate and severe stertor sounds is that they can be heard without a stethoscope [10]. However, this study’s sound recordings were made using a stethoscope. The sounds recorded at the larynx will have different frequency responses than those heard by a human ear in free air, so it may be challenging to replicate the stertor label in some cases. Another source of discrepancy is that the stertor label was assigned by veterinarians listening to the sounds, so some subjective variation is possible, particularly between different annotators.

The sensitivity and specificity of the algorithm for distinguishing positive and negative BOAS based on stertor are promising for improving the accessibility of diagnosis. Even though the model was not directly trained to predict BOAS grade, its performance on it is marginally better than that of the original stertor task. This may be due to an ensemble effect from averaging multiple recordings’ predictions, which removes spurious errors. The performance of this ground-truth label at predicting the final functional grade of BOAS is excellent, justifying the training of a stertor model to predict functional grade. However, a limitation of this dataset is its size, resulting in larger confidence intervals in the results as expected.

The per-class results in Table 3 demonstrate that the model struggles to predict the individual BOAS functional grades accurately. Examination of individual confusion matrices shows that the grade 0 cases are often misdiagnosed as grade 1, whilst the grade 3 cases are often misdiagnosed as grade 2. This performance can be attributed to two reasons. Firstly, the dataset is small, with most cases in grade 1 or 2. The nature of recruitment, predominantly in hospital referral, limits the number of pure control cases in the dataset. This small number of examples makes accurate training of a neural network challenging and increases the risk of overfitting. Secondly, distinguishing between individual grades in the RFG scheme based purely on stertor is a challenging task. The presence of dyspnoea, cyanosis and significant respiratory effort are key indicators that will result in a grade 3 BOAS patient but will not necessarily have audible signs in the recording. Given that the final BOAS grade is not a continuous scale, there will be some overlap between adjacent grades when considering individual predictive features such as stertor.

A further limitation of the model is that it was not trained to predict the presence of stridor, and as a result some significant BOAS cases were missed. The number of examples of stridor was limited (see Table 2), which meant that training a machine learning model to identify it was infeasible. However, stridor can be a sensitive indicator of pathology. In the French bulldog and bulldog, as stridor is audible in severely affected dogs, it may distinguish between grade 2 and 3 dogs. In the pug, a minority of dogs have predominantly stridor rather than stertor supportive of a primary laryngomalacia; so future research should focus on collecting more examples of stridor sounds.

This analysis was performed retrospectively using data collected by experienced clinicians, often in a hospital environment. This means that there will be an inevitable distribution shift in the data when deployed clinically. Recording quality may be reduced due to poor placement, excess stethoscope movement and environmental noise. Therefore, additional signal quality assessment stages in the algorithm may be required to inform the user when a signal is too noisy to analyse. Future trials of the algorithm should take place in the desired clinical environment, with the performance of the algorithm then compared to human predictions to assess its efficacy.

## Conclusions

This research has applied machine learning to automate the analysis of brachycephalic respiratory sounds, with the aim of making the functional grading of BOAS accurate and repeatable for a non-expert. The growing popularity of brachycephalic breeds means widespread community early detection of BOAS is essential for timely intervention and breeding decisions. The solution proposed here only requires an electronic stethoscope placed by a user at the larynx, with an algorithm replicating an expert’s interpretation and grading of sounds. Combined with other variables from the RFG scheme, this may enable general veterinarians, nurses and even owners to detect signs of clinically significant BOAS.

The presented recurrent neural network achieved promising results, with an accuracy well within the range of existing screening tests for humans, despite a small and challenging dataset that included considerable noise and artefacts. These algorithms serve as a proof of concept that machine learning may be used to automatically produce a clinically significant grading of BOAS. Future research will focus on collecting greater quantities of data in representative clinical environments, enabling better neural network training. Identification and grading of the high-frequency stridor sound will also increase the sensitivity of the algorithm, particularly in key severe cases. The predictions of stertor and stridor could then be combined with other parameters from the RFG scheme to provide a robust screening technique for BOAS.

In clinical practice, the algorithm could be deployed as part of a smartphone application that links to an electronic stethoscope wirelessly to record the sounds. Electronic stethoscopes are increasingly popular in the medical and veterinary world, offering Bluetooth functionality to record and transmit sounds. The app could provide basic training on how to connect and record with an electronic stethoscope, providing simple instructions to limit the need for specialised training. Use of an electronic stethoscope by owners may require some training on auscultation and the feasibility of this should be investigated further. Alternatively, acoustic sensors could be integrated into a collar for more widespread use by owners, allowing them to track the progression of the disease accurately and objectively at home. If analysis of the sounds predicts clinically significant BOAS, the app could then refer owners to veterinarians for further assessment and intervention.
